# Genomic profiling identifies common HPV-associated chromosomal alterations in squamous cell carcinomas of cervix and head and neck

**DOI:** 10.1186/1755-8794-2-32

**Published:** 2009-06-01

**Authors:** Saskia M Wilting, Serge J Smeets, Peter JF Snijders, Wessel N van Wieringen, Mark A van de Wiel, Gerrit A Meijer, Bauke Ylstra, C René Leemans, Chris JLM Meijer, Ruud H Brakenhoff, Boudewijn JM Braakhuis, Renske DM Steenbergen

**Affiliations:** 1 Department of Pathology, VU University Medical Center, Amsterdam, The Netherlands; 2 Department of Otolaryngology/Head-Neck Surgery, VU University Medical Center, Amsterdam, The Netherlands; 3 Department of Epidemiology and Biostatistics, VU University Medical Center, Amsterdam, The Netherlands

## Abstract

**Background:**

It is well known that a persistent infection with high-risk human papillomavirus (hrHPV) is causally involved in the development of squamous cell carcinomas of the uterine cervix (CxSCCs) and a subset of SCCs of the head and neck (HNSCCs). The latter differ from hrHPV-negative HNSCCs at the clinical and molecular level.

**Methods:**

To determine whether hrHPV-associated SCCs arising from different organs have specific chromosomal alterations in common, we compared genome-wide chromosomal profiles of 10 CxSCCs (all hrHPV-positive) with 12 hrHPV-positive HNSCCs and 30 hrHPV-negative HNSCCs. Potential organ-specific alterations and alterations shared by SCCs in general were investigated as well.

**Results:**

Unsupervised hierarchical clustering resulted in one mainly hrHPV-positive and one mainly hrHPV-negative cluster. Interestingly, loss at 13q and gain at 20q were frequent in HPV-positive carcinomas of both origins, but uncommon in hrHPV-negative HNSCCs, indicating that these alterations are associated with hrHPV-mediated carcinogenesis. Within the group of hrHPV-positive carcinomas, HNSCCs more frequently showed gains of multiple regions at 8q whereas CxSCCs more often showed loss at 17p. Finally, gains at 3q24-29 and losses at 11q22.3-25 were frequent (>50%) in all sample groups.

**Conclusion:**

In this study hrHPV-specific, organ-specific, and pan-SCC chromosomal alterations were identified. The existence of hrHPV-specific alterations in SCCs of different anatomical origin, suggests that these alterations are crucial for hrHPV-mediated carcinogenesis.

## Background

In the pathogenesis of uterine cervical cancer the necessary and causal involvement of high-risk types of the human papillomavirus (hrHPV) is widely accepted and supported by strong epidemiological and molecular evidence [1]. HrHPV is present in virtually all cervical carcinomas and the viral oncogenes E6 and E7 are consistently expressed in cervical cancers and precancers. Deregulated expression of these oncogenes in the basal, dividing cells of the epithelium interferes with cell cycle control due to their ability to induce degradation of the tumour suppressor proteins p53 and pRb, respectively. This results in uncontrolled cell proliferation and accumulation of specific (epi)genetic changes in the host cell genome, driving progression to a malignant phenotype [2,3]. In a previous study we have used array-based comparative genomic hybridisation (array CGH) to determine frequent chromosomal alterations in cervical cancer, which included gains at 1q, 3q and 20q and losses at 8q, 10q, 11q, and 13q [4]. The necessity of these and other additional (epi)genetic alterations in the carcinogenic process is illustrated by the fact that their frequency increases with increasing severity of cervical disease.

On the other hand, head and neck squamous cell carcinoma (HNSCC) is known to be mainly caused by well-established life-style related habits, such as tobacco and excessive alcohol consumption. However, besides the influence of these life-style carcinogens, high risk human papillomavirus (hrHPV) is present in 15–35% of HNSCCs and has been suggested to be a separate aetiological factor in head-and-neck carcinogenesis [5-8]. Several studies have shown that hrHPV-positive HNSCCs are associated with a better clinical outcome [7-9]. Moreover, molecular differences were found between hrHPV-positive and hrHPV-negative HNSCCs, supporting the idea of two separate carcinogenic pathways to HNSCC, one determined by life-style carcinogens and the other by hrHPV [5,6,10-13].

In a previous study, using array CGH, we identified a number of chromosomal alterations specific for hrHPV-negative HNSCCs that were absent in hrHPV-positive HNSCCs, including loss at 3p, 5q, and 9p, and amplifications at 11q [13]. The hrHPV-positive HNSCCs were characterised by a lower level of chromosomal alterations, none of which were hrHPV-specific. To investigate potential organ-independent, hrHPV-associated chromosomal alterations, genomic profiles of cervical SCCs (CxSCCs), hrHPV-positive and hrHPV-negative HNSCCs were compared using sophisticated clustering and statistical approaches [4,13]. In addition, we also investigated the presence of organ-specific alterations and alterations shared by all SCCs included in this study.

## Methods

### CGH microarrays

We used chromosomal profiles of 30 hrHPV-negative HNSCCs, 12 hrHPV-positive HNSCCs and 10 CxSCCs all of which were previously described (Table 1) [4,13,14]. To avoid possible confounding of our results, we excluded a subset of hrHPV-negative HNSCCs described in Smeets *et al*, which showed little to no alterations and should therefore be considered a separate group [14].

**Table 1:** Summary of clinical data of carcinomas included in this study.

**Sample ID**	**origin**	**hrHPV (+/-)**	**age (yrs)**	**sex**
CxSCC2	cervix	+	39	female
CxSCC4	cervix	+	62	female
CxSCC12	cervix	+	44	female
CxSCC15	cervix	+	47	female
CxSCC27	cervix	+	49	female
CxSCC28	cervix	+	48	female
CxSCC32	cervix	+	37	female
CxSCC36	cervix	+	72	female
CxSCC38	cervix	+	51	female
CxSCC39	cervix	+	40	female

HNSCC9741	oropharynx	-	53	female
HNSCC9762	oral cavity	-	76	female
HNSCC9773	oral cavity	-	49	male
HNSCC9830	oral cavity	-	42	male
HNSCC9848	oropharynx	-	59	male
HNSCC9892	oral cavity	-	67	male
HNSCC9897	oral cavity	-	72	male
HNSCC9902	oral cavity	-	53	female
HNSCC9942	oral cavity	-	55	female
HNSCC9952	oral cavity	-	61	female
HNSCC9956	oral cavity	-	38	male
HNSCC2014	oral cavity	-	68	female
HNSCC2034	oral cavity	-	45	female
HNSCC9738	oral cavity	-	60	male
HNSCC9745	oropharynx	-	52	male
HNSCC9750	oral cavity	-	65	male
HNSCC9812	oral cavity	-	65	female
HNSCC9827	oropharynx	-	57	male
HNSCC9829	oral cavity	-	74	female
HNSCC9831	oral cavity	-	55	male
HNSCC9841	oropharynx	-	55	female
HNSCC9847	oropharynx	-	57	male
HNSCC9880	oral cavity	-	71	female
HNSCC9907	oral cavity	-	46	male
HNSCC9914	oropharynx	-	78	male
HNSCC9926	oropharynx	-	60	male
HNSCC9957	oral cavity	-	51	male
HNSCC9970	oral cavity	-	49	female
HNSCC9981	oropharynx	-	55	female
HNSCC9987	oral cavity	-	79	male

HNSCC9881	oral cavity	+	51	male
HNSCC9729	oropharynx	+	52	male
HNSCC9838	oropharynx	+	60	male
HNSCC9860	oropharynx	+	67	female
HNSCC9901	oropharynx	+	57	female
HNSCC9948	oropharynx	+	70	male
HNSCC9951	oropharynx	+	46	male
HNSCC9808	oral cavity	+	46	male
HNSCC9859	oral cavity	+	40	male
HNSCC9924	oral cavity	+	41	female
HNSCC9947	oral cavity	+	72	male
HNSCC2015	oral cavity	+	65	male

CGH BAC microarrays produced at the Microarray facility of the VU Medical Center were used. These arrays included the 1 Mb resolution Sanger BAC clone set and a subset of clones from the Children's Hospital Oakland Research Institute (CHORI). Spots were quantified using ImaGene 5.6.1 software (BioDiscovery Ltd, Marina del Rey, CA, USA) with default settings for the flagging of bad quality spots.

The entire dataset described here is available from the Gene Expression Omnibus (GEO, ) through series accession numbers GSE6473 (CxSCC) and GSE12020 (HNSCC).

This study followed the ethical guidelines of the Institutional Review Board of the VU University Medical Center and informed consent was obtained from all patients included.

### Array CGH analysis

#### Calling of gains and losses

BAC clones were positioned along the genome according to the May 2004 freeze. After exclusion of clones with one or more flagged spots, the average of the triplicate spots was calculated for each BAC clone. Log_2 _ratios were normalised per spotted sub-array by subtraction of the median value of all BAC clones spotted within that sub-array. Segmentation and subsequent calling of gained, amplified and lost regions was done using CGHCall, an automated calling algorithm. Segments with a probability score of ≥ 0.5 were considered gained, amplified or lost [15].

#### Reduction of dataset into chromosomal regions

We used the CGHregions algorithm to reduce our dataset to chromosomal regions, accepting maximally 0.1% information loss (Threshold = 0.001). It was shown by Van de Wiel *et al *that the use of regions instead of single BAC clones improved the effectiveness of subsequent statistical analyses and facilitated interpretation of the results [16].

#### Clustering analysis

The samples were clustered by means of a modified version of WECCA [17]. WECCA is a hierarchical clustering method tailor-made for called aCGH data. The modified version accommodates the use of call probabilities instead of calls. The use of the call probabilities in the unsupervised analysis will give a more subtle picture of the similarities and differences between the samples. The modified version of WECCA defines the distance between two features as the symmetric Kullback-Leibler divergence. The distance between the call probability profiles of two samples is then defined as the average of these divergences over all features. In the construction of the dendrogram we used Ward's linkage as it yields compact and well-separated clusters.

### Statistical analysis

The association between clustering results and HPV status was determined by chi-square testing. The average total number of altered regions was compared between HPV-positive and HPV-negative carcinomas using the non-parametric Mann Whitney test. Two-sided p-values below 0.05 were considered statistically significant. Alteration patterns between HPV-positive (HNSCCs and CxSCCs) and HPV-negative tumours as well as between HNSCCs and CxSCCs were compared using a binomial differential proportion test. The test procedure includes a permutation-based false discovery rate (FDR) correction for multiple testing, needed to discriminate real differences from chance effects [18]. An FDR below 0.10 was considered statistically significant.

### Gene ontology analysis

To interpret the biological significance of the genes that are located at altered chromosomal regions of interest, a gene ontology analysis was performed using Ingenuity Pathways Analysis (Ingenuity Systems^®^, Redwood City, USA). Biological/molecular functions were considered to be significantly overrepresented if they contained more than 1 gene and the Benjamini-Hochberg corrected p-value was p < 0.10 [19].

## Results

### hrHPV-positive carcinomas cluster together

To obtain an overview of the similarities between samples, unsupervised hierarchical clustering was performed. This method enabled us to determine in an unbiased manner whether chromosomal profiles of hrHPV-positive HNSCCs were more closely related to hrHPV-negative HNSCCs or hrHPV-positive CxSCCs.

**Figure 1:**
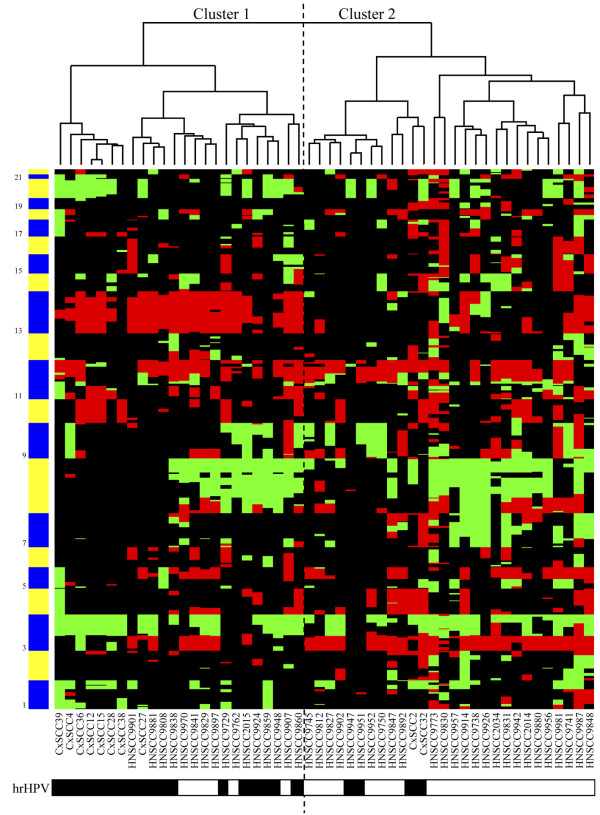
**Result from the unsupervised hierarchical clustering analysis**. Cluster 1 contains in majority hrHPV-positive carcinomas, as is indicated by the black boxes in the legend underneath the heatmap (p < 0.0001).

As is shown in Figure 1, two clusters emerged. Cluster 1 contained 24 samples, 18 of which were hrHPV-positive (75%) and 6 were hrHPV-negative. Cluster 2 contained 28 samples of which 4 samples were hrHPV-positive and 24 were hrHPV-negative (86%). This association between cluster assignment and hrHPV status was statistically significant (p < 0.0001). The hrHPV-positive cluster 1 included both samples of cervical (n = 8) and head and neck origin (n = 10), indicating similarities between the chromosomal profiles of hrHPV-induced carcinomas of different anatomical origins. Within cluster 1, however, 7 out of 8 cervical samples formed a separate sub-cluster. This suggests that organ-specific alterations exist as well in hrHPV-positive HNSCCs and CxSCCs.

### hrHPV-associated loss at chromosome 13q and gain at 20q

**Figure 2:**
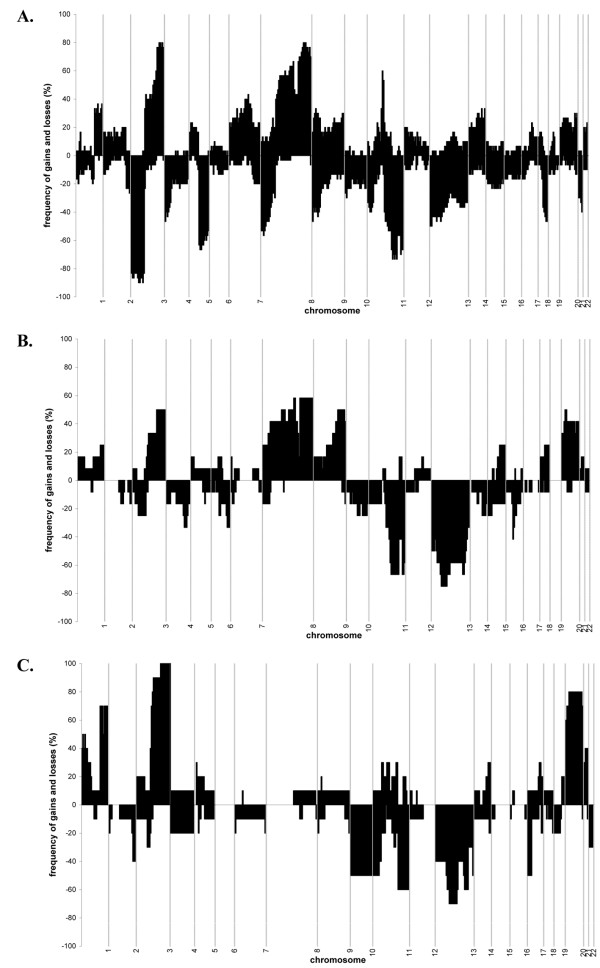
**Frequency plots for all 3 sample groups**. The frequency of gains (positive axis) and losses (negative axis) are shown for **A**. hrHPV-negative HNSCCs, **B**. **hr**HPV-positive HNSCCs and **C**. CxSCCs for chromosome 1–22.

To assess the differences between all three sample groups, the frequency of gains (including amplifications) and losses was analysed for all chromosomal regions (Figure 2). In general, hrHPV-negative carcinomas showed significantly more altered regions than hrHPV-positive carcinomas (p = 0.022).

To determine hrHPV-specific chromosomal alterations, the frequency of alterations was compared between hrHPV-positive (12 HNSCCs and 10 CxSCCs) and hrHPV-negative tumours (30 HNSCCs) for all chromosomal regions. Regions showing a significant difference (FDR < 0.10) in this comparison are shown in Table 2. Interestingly, loss of 13q21.1-21.33 and gain of 20p12.1-q13.33 were significantly more frequent in HPV-positive SCCs compared to hrHPV-negative HNSCCs (66.7% and 58.5% in hrHPV-positive SCCs compared to 33% and 24% in hrHPV-negative SCCs, respectively). As was also shown in our previous study, loss of regions at 3p and 5q, and gains/amplifications of a small region at 11q (CCND1 locus) were specific for hrHPV-negative HNSCCs [13]. A region at 8p showed loss in hrHPV-negative HNSCCs, gain in hrHPV-positive HNSCCs and no alteration in CxSCCs. Gain at chromosome 8q was more frequent in hrHPV-negative HNSCCs, which was due to absence of this alteration in CxSCCs.

**Table 2:** Significantly different chromosomal alterations between hrHPV+ and hrHPV- carcinomas.

					**HPV+ (HNSCC; CxSCC)**			**HPV-**		
**chromosome**	**Start (Mb)**	**End (Mb)**	**Cytoband**	**FDR**	**% loss**	**% gain**	**% amp**	**% loss**	**%gain**	**%amp**
1	1.08	58.69	1p36.33-p32.2	0.08	0.0	26.7 (15.6; 40.0)	0.0	14.6	5.8	0.0
1	109.78	117.23	1p13.3-p13.1	0.04	0.0	13.6 (16.7; 10.0)	0.0	17.8	0.0	0.0
2	96.17	97.68	2q11.2	0.08	4.5 (0.0; 10.1)	0.0	0.0	0.0	20.0	0.0
2	165.89	180.53	2q24.3-q31.3	0.06	9.1 (8.3; 10.0)	0.0	0.0	0.0	18.9	0.0
*3*	*0.28*	*86.18*	*3p26.3-p12.1*	*0.00*	*17.8 (20.5; 14.6)*	*9.4 (3.8; 16.2)*	*0.0*	*86.2*	*0.5*	*0.0*
*5*	*51.16*	*180.57*	*5q11.2-q35.3*	*0.00*	*8.6 (7.5; 10.0)*	*9.1 (8.3; 10.0)*	*0.0*	*61.0*	*0.3*	*0.0*
7	9.95	25.67	7p21.3-p15.2	0.05	13.6 (16.7; 10.0)	0.0	0.0	3.3	21.7	0.0
7	65.52	116.45	7q11.21-q31.2	0.03	4.5 (0.0; 10.0)	0.3 (0.6; 0.0)	0.0	4.1	32.1	2.8
8	0.38	34.51	8p23.3-p12	0.02	6.8 (12.5; 0.0)	17.3 (31.7; 0.0)	0.0	48.7	12.0	0.0
8	75.65	93.93	8q21.11-q22.1	0.06	0.0	22.7 (41.7; 0.0)	0.0	3.3	57.8	0.0
8	114.72	145.68	8q23.3-q24.3	0.05	0.3 (0.0; 0.7)	34.4 (55.4; 9.3)	0.0	0.5	70.2	2.1
*11*	*69.18*	*70.31*	*11q13.3-q13.4*	*0.04*	*11.4 (12.5; 10.0)*	*13.6 (0.0; 30.0)*	*4.5 (8.3; 0.0)*	*10.0*	*15.0*	*41.7*
**13**	**55.72**	**71.49**	**13q21.1-q21.33**	**0.06**	**66.7 (64.8; 68.9)**	**0.0**	**0.0**	**33.0**	**7.0**	**2.2**
14	47.08	61.97	14q21.3-q23.2	0.08	11.4 (12.5; 10.0)	2.3 (0.0; 5.0)	0.0	8.3	28.3	0.0
14	79.43	86.91	14q31.1-q31.3	0.09	18.2 (25.0; 10.0)	9.1 (0.0; 20.0)	0.0	6.7	33.3	0.0
18	28.34	75.62	18q12.1-q23	0.03	10.0 (8.3; 12.0)	18.2 (25.0; 10.0)	0.0	42.7	2.0	0.0
**20**	**15.56**	**60.28**	**20p12.1-q13.33**	**0.06**	**1.5 (2.8; 0.0)**	**58.5 (41.7; 78.7)**	**0.0**	**6.2**	**24.0**	**0.0**
21	14.68	46.85	21q11.2-q22.3	0.00	1.1 (0.0; 2.5)	25.0 (14.6; 37.5)	0.0	33.3	2.5	0.0

hrHPV-specific alterations are printed in bold. Alterations specific for hrHPV-negative HNSCCs are italicised. Mb; Megabases, FDR; False Discovery Rate, amp; amplification

Our unsupervised classification results indicated that, within our hrHPV-positive cluster, CxSCCs formed a separate group. To identify potential organ-specific alterations, we therefore also compared the frequency of alterations for all regions between hrHPV-positive HNSCCs (n = 12) and CxSCCs (n = 10) (Table 3). Gains at 3q and losses at 17p were significantly more frequent in CxSCCs than HNSCCs (FDR < 0.10). On the other hand, HNSCCs showed frequent gains at chromosome 8q and losses at 11q. It is important to note that even though gain at 3q and loss at 11q were significantly different between hrHPV-positive HNSCCs and CxSCCs, these alterations were frequent in all sample groups (>50%). Significant differences for these regions can mainly be explained by the fact that the size of the exact altered region differed between groups. Therefore, the smallest regions of overlap between all samples, namely 3q24-29 and 11q22.3-25, may represent general alterations in carcinomas derived from squamous epithelium.

**Table 3:** Significantly different chromosomal alterations between hrHPV-positive HNSCCs and CxSCCs.

					**HNSCC+**			**CxSCC**		
**chromosome**	**start (Mb)**	**end (Mb)**	**Cytoband**	**FDR**	**% loss**	**% gain**	**% amp**	**% loss**	**%gain**	**% amp**
3	112.84	199.07	3q13.2-q29	0.09	0.0	40.6	2.1	0.0	95.6	0.0
**8**	**61.67**	**74.46**	**8q12.1-q21.11**	**0.09**	**2.1**	**50.0**	**0.0**	**0.0**	**0.0**	**0.0**
**8**	**94.15**	**99.29**	**8q22.1-q22.2**	**0.09**	**0.0**	**50.0**	**0.0**	**0.0**	**0.0**	**0.0**
**8**	**142.29**	**145.68**	**8q24.3**	**0.09**	**0.0**	**58.3**	**0.0**	**10.0**	**0.0**	**0.0**
11	98.40	105.02	11q22.1-q22.3	0.09	66.7	0.0	0.0	20.0	30.0	0.0
*17*	*0.91*	*2.40*	*17p13.3*	*0.09*	*0.0*	*0.0*	*0.0*	*50.0*	*10.0*	*0.0*
*17*	*19.19*	*19.29*	*17p11.2*	*0.09*	*0.0*	*0.0*	*0.0*	*50.0*	*10.0*	*0.0*

Alterations specific for HNSCCs are printed in bold, alterations specific for CxSCCs are italicised. Mb;Megabases, FDR; False Discovery Rate, amp; amplification

**Figure 3:**
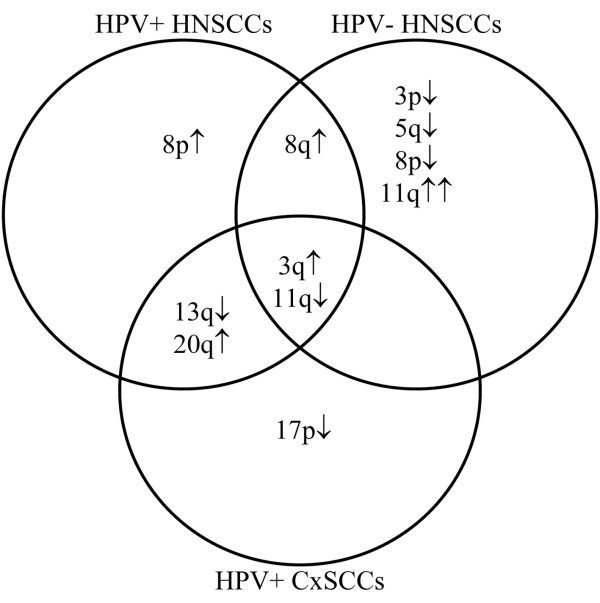
**Summary of common and specific chromosomal alterations in the different sample groups**. Chromosome arms showing frequent alterations (>50%) in one or more sample groups (hrHPV-positive HNSCCs; hrHPV-negative HNSCCs; CxSCCs) are shown in a Venn diagram. ↑ indicates gain; ↓ indicates loss

All results described above are summarised in a Venn diagram, showing a general overview of the frequently altered chromosomal arms specific to or common between the (sub) groups (Figure 3).

### Biological functions of genes located within hrHPV-specific chromosomal alterations

**Figure 4:**
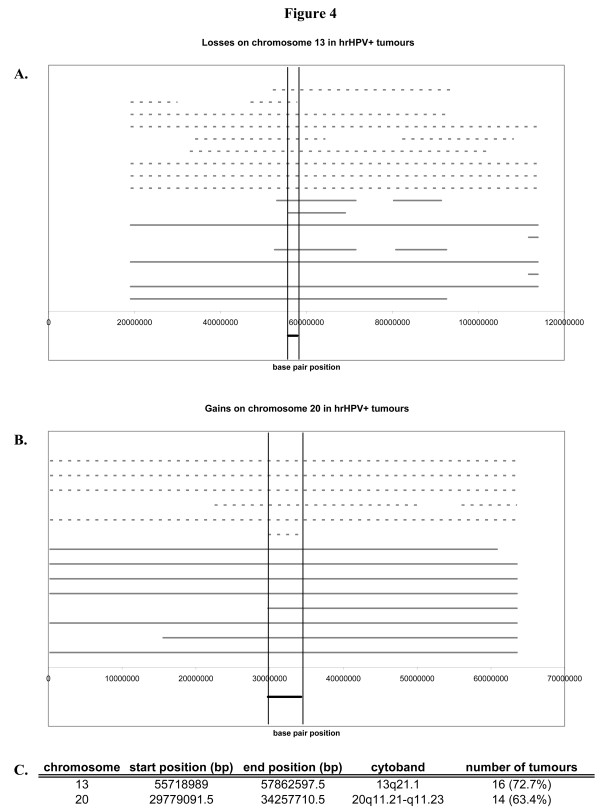
**Genomic coordinates of A. losses at chromosome 13 and B. gains at chromosome 20 are shown for all hrHPV-positive carcinomas**. Chromosomal alterations in CxSCCs are shown by dashed lines and alterations in hrHPV-positive HNSCCs by solid lines. In **C**. the smallest regions of overlap (SROs) between hrHPV-positive carcinomas at chromosome 13 and 20 are summarised.

As described above gain at 20p12.1-q13.33 and loss at 13q21.1-21.33 were significantly more frequent in hrHPV-positive SCCs compared to hrHPV-negative ones. In fact, a 4.5 megabase (Mb) region on chromosome 20 (20q11.21-q11.23), and a 2 Mb region on chromosome 13 (13q21.1) formed the smallest regions of overlap (SRO) at these respective loci when all hrHPV-positive carcinomas were considered (Figure 4A and 4B). The SRO at chromosome 20q contains 78 genes and the one at chromosome 13q contains 6 genes [see Additional file 1]. Within the SRO at chromosome 20, two genes reside, i.e. NCOA6 and RBM39, which showed elevated expression in hrHPV16 E7 expressing cells *in vitro *[20]. Other cancer-related genes located within this SRO include PIGU, E2F1, and DNMT3B. The SRO on chromosome 13 encompasses the PCDH17 gene and a cluster of five identical loci all of which are predicted to encode proline-rich proteins that contain several dopamine D4 receptor signatures.

Subsequent gene ontology analysis of all genes located in these SROs identified a number of significantly overrepresented GO biological/molecular functions, including cell cycle, cell-to-cell signalling and interaction, cellular growth and proliferation, and a number of cellular maintenance functions (i.e. DNA replication, recombination and repair, gene expression, cellular function and maintenance, cellular assembly and organisation, cellular compromise, cell death, cell morphology, cellular development, nucleic acid metabolism, and lipid metabolism).

## Discussion

In the present study we compared genome-wide chromosomal profiles of hrHPV-negative HNSCCs, hrHPV-positive HNSCCs and hrHPV-positive CxSCCs, to determine whether hrHPV-associated carcinomas of different origins have similar chromosomal signatures. In addition, potential organ-specific alterations were determined within the total group of hrHPV-positive SCCs.

Unsupervised hierarchical clustering resulted in a separate hrHPV-positive cluster, indicating similarities in the chromosomal profiles of hrHPV-induced carcinomas. Subsequent supervised statistical analysis identified a number of hrHPV-associated chromosomal alterations, including gains at 20p13-q13.33 and losses at 13q21.1-21.33, which were frequent (>50%) in hrHPV-positive carcinomas, but were only rarely observed in hrHPV-negative carcinomas. In contrast, lost regions at 3p and 5q as well as amplifications at 11q13.3 (CCND1 locus) were frequent in hrHPV-negative carcinomas, but not in hrHPV-positive carcinomas. In our previous study comparing only hrHPV-positive and hrHPV-negative HNSCCs, the same regions were identified as well as being specific for hrHPV-negative HNSCCs [13].

In the present study we found that gains at 20q and losses at 13q are specific for hrHPV-positive carcinomas of different anatomical origins. Our previous study, including only hrHPV-positive and hrHPV-negative HNSCCs, did not identify these alterations as specific for hrHPV-positive HNSCCs. However, in the present study a larger number of hrHPV-negative hrHNSCCs and HPV-positive SCCs was included. Furthermore, a sophisticated, objective calling method was presently used to determine gained and lost regions [15]. Interestingly, in a number of studies describing hrHPV E6 and/or E7 mediated immortalisation of keratinocytes of multiple anatomical origins, gains of chromosome 20q and losses of chromosome 13q were consistently observed [20-23]. Low-level gains of chromosome 20q are suggested to be caused by E7 expression and consequent inactivation of the pRb pathway in epithelial cells [20,21,24]. Deletion of part of the long arm of chromosome 13 is frequently found in a number of hrHPV-associated tumours, including cervical, anal and head and neck carcinomas [4,13,25]. Sabbir *et al *showed that loss of 13q in HNSCCs was associated with the presence of hrHPV, which is in agreement with our findings [26].

In a recent study Pyeon *et al *showed that the gene expression patterns of hrHPV-positive HNSCCs and CxSCCs differed yet shared many changes compared to hrHPV-negative HNSCCs [10]. Our study shows that the same holds true on a chromosomal level. Interestingly, 28% of the genes Pyeon *et al *found to be differentially expressed between hrHPV-positive and hrHPV-negative carcinomas is located within the chromosomal regions identified in this study and showed expression changes concordant with the chromosomal alterations. Of these genes, 39% was located at chromosome 1p, 25% at 5q, and 14% at 3p. The other genes were located at 11q, 18q and 21q. Only one gene, SYCP2, was located within the hrHPV-specific chromosomal alterations found in this study (20q), but was not located within our SRO. To the best of our knowledge none of the genes are known to directly interact with hrHPVE6 and/or E7 [27,28]. Pathway analysis of all genes overlapping with our findings identified cell cycle/proliferation as most overrepresented biological function, which is in concordance with the observations made by Pyeon *et al *[10].

Pathway analysis of all genes located within the hrHPV-associated SROs at chromosome 20q and 13q found in this study, again underlined the importance of cell cycle (replication and proliferation) related genes in hrHPV-mediated carcinogenesis. This may be related to the continuous E7-regulated E2F1 activation and is accompanied by changes in overall cellular maintenance systems, such as nucleic acid metabolism, as was also found in HPV16 E7 expressing epithelial cells *in vitro *[20]. Two genes, NCOA6 and RBM39, overlapped between this *in vitro *study and our results, warranting further investigation of their role in hrHPV-mediated transformation. NCOA6 encodes a transcriptional coactivator interacting with basal transcription factors, histone acetyltransferases, and methyltransferases. RBM39 encodes an RNA binding protein and possible splicing factor and may act as a transcriptional coactivator for the AP-1 transcription activator complex and estrogen receptors. Other cancer-related genes at 20q include E2F1, which is specifically targeted by hrHPV-mediated degradation of pRb, PIGU, which may play a role in cell cycle control and was identified as an oncogene in bladder cancer [29], and DNMT3B, a de novo DNA methyl transferase. We previously showed that DNMT3B is amplified in the cervical cancer cell line SiHa and found a correlation between increased DNMT3B gene copy numbers and elevated mRNA expression in 78% of CxSCCs [4]. The increased DNMT3B levels are most likely related to the high frequency of tumour suppressor gene promoter hypermethylation events during hrHPV-mediated carcinogenesis [30-32]. The SRO at chromosome 13q includes one known gene, PCDH17, a member of the protocadherin family, which is a subfamily of the cadherin superfamily. At present no reports are available describing interactions between hrHPV and protocadherins. However hrHPV presence has been related to decreased E-cadherin and subsequent impaired immune response [33-35].

Next to the hrHPV-related common events, organ-specific alterations for CxSCCs and HNSCCs were identified as well. CxSCCs more frequently showed loss at 17p, while HNSCCs were characterised by frequent gains at chromosome 8q, harbouring the oncogene c-Myc [36].

Gains at 3q and losses at 11q were found frequently in all SCCs included in this study, suggesting their involvement in carcinogenesis of squamous epithelial cells in general. Interestingly, we previously showed that gains of 3q were highly frequent in CxSCCs but not in adenocarcinomas of the same organ, further emphasising that this alteration may be specific for squamous epithelium [4].

## Conclusion

Together with results from previous studies, our findings support a causal role for hrHPV in the development of a subset of HNSCCs. Consequently, hrHPV-positive and hrHPV-negative HNSCCs should be regarded as different disease entities requiring different diagnostic and therapeutic approaches. The fact that hrHPV-associated SCCs of different organs have chromosomal alterations in common, suggests that these alterations are crucial for hrHPV-induced carcinogenesis. Diagnostic and/or therapeutic targets based on these alterations may therefore be relevant to hrHPV-associated SCCs of all anatomical origins.

## Abbreviations

hrHPV: high-risk human papillomavirus; CxSCC: squamous cell carcinoma of the uterine cervix; HNSCC: squamous cell carcinoma of the head and neck; SCC: squamous cell carcinoma; CGH: comparative genomic hybridisation; BAC: bacterial artificial chromosome; Mb: megabase; FDR: false discovery rate; SRO: smallest region of overlap; GO: gene ontology.

## Competing interests

The authors declare that they have no competing interests.

## Authors' contributions

SMW and SJS performed array experiments and data analysis and drafted the manuscript. RDMS and BJMB participated in the design of the study and the drafting of the manuscript. WNvW and MAvdW contributed to the statistical analyses. BY and GAM were involved in the development of the micorarray platform and provided the facilities for the microarray experiments. PJFS, CRL, CJLMM and RHB contributed to the conception of the study and critically revised the manuscript. All authors read and approved the final manuscript.

## Pre-publication history

The pre-publication history for this paper can be accessed here:



## Supplementary Material

Additional file 1 **BAC clones and genes included in the SROs of the hrHPV-specific chromosomal alterations at chromosomes 13q and 20q**. All BAC clones and genes located within the identified SROs at chromosome 20q and 13q are listed here. Click here for file
