# Supplementary material for: Genomic profiling identifies common HPV-associated chromosomal alterations in squamous cell carcinomas of cervix and head and neck
Source: BMC Med Genomics. 2009 Jun 1;2:32. doi: 10.1186/1755-8794-2-32 (PMC2698908; doi:10.1186/1755-8794-2-32)
Supplement: Additional file 1 — BAC clones and genes included in the SROs of the hrHPV-specific chromosomal alterations at chromosomes 13q and 20q. All BAC clones and genes located within the identified SROs at chromosome 20q and 13q are listed here. [file 1755-8794-2-32-S1.doc]

| **Cytoband** | **Start position (bp)** | **End position (bp)** | **BAC clones** | **Genes** |
| --- | --- | --- | --- | --- |
| 20q11.21-q11.23 | 29779091.5 | 34257710.5 | RP5-857M17; RP1-310O13; RP5-854E16; RP11-410N8; RP5-1018D12; RP5-1093G12; RP5-1085F17; RP4-733O23; RP5-1137F22; RP5-1184F4; RP4-553F4; RP1-18C9; RP11-234K24 | TPX2; MYLK2; FOXS1; DUSP15; TTLL9; PDRG1; XKR7; C20orf160; HCK; TM9SF4; TSPYL3; PLAGL2; POFUT1; KIF3B; ASXL1; C20orf112; LOC284805; COMMD7; DNMT3B; MAPRE1; EFCAB8; SPAG4L; BPIL1; BPIL3; C20orf185; C20orf186; C20orf70; BASE; C20orf71; PLUNC; C20orf114; CDK5RAP1; SNTA1; CBFA2T2; NECAB3; C20orf144; C20orf134; E2F1; PXMP4; ZNF341; CHMP4B; RALY; EIF2S2; ASIP; AHCY; ITCH; DYNLRB1; MAP1LC3A; PIGU; TP53INP2; NCOA6; HMGB3L1; GGT7; ACSS2; GSS; MYH7B; TRPC4AP; EDEM2; PROCR; MMP24; EIF6; FAM83C; UQCC; GDF5; CEP250; C20orf173; ERGIC3; FER1L4; SPAG4; CPNE1; RBM12; NFS1; ROMO1; RBM39; PHF20; SCAND1; C20orf152; EPB41L1 |
| 13q21.1 | 55718989 | 57862597.5 | RP11-640E11; RP11-205J24; RP11-516G5; RP11-204N9; RP11-435P18; RP11-98F3; RP11-111C7; RP11-522F22; RP11-334O13; RP11-538C21; RP11-168J5 | LOC729233; LOC729240; LOC729246; LOC729250; PRR20; PCDH17 |
